# Biomarkers to Predict Multiorgan Distress Syndrome and Acute Kidney Injury in Critically Ill Surgical Patients

**DOI:** 10.3390/medicina59122054

**Published:** 2023-11-21

**Authors:** In Sik Shin, Da Kyung Kim, Sanghyun An, Sung Chan Gong, Moo Hyun Kim, Md Habibur Rahman, Cheol-Su Kim, Joon Hyeong Sohn, Kwangmin Kim, Hoon Ryu

**Affiliations:** 1 Division of Acute Care Surgery, Department of Surgery, Yonsei University Wonju College of Medicine, Wonju 26426, Republic of Korea; fleece2@yonsei.ac.kr (I.S.S.); kmh3789@yonsei.ac.kr (M.H.K.); 2 Yonsei University Wonju College of Medicine, Wonju 26426, Republic of Korea; judi_kim@naver.com; 3 Division of Colorectal Surgery, Department of Surgery, Yonsei University Wonju College of Medicine, Wonju 26426, Republic of Korea; uldura@yonsei.ac.kr; 4 Center of Evidence Based Medicine, Institute of Convergence Science, Yonsei University, Seoul 03722, Republic of Korea; 5 Division of Esophago-Gastrointestinal Surgery, Department of Surgery, Yonsei University Wonju College of Medicine, Wonju 26426, Republic of Korea; surgeon_g@yonsei.ac.kr; 6 Department of Convergence Medicine, Yonsei University Wonju College of Medicine, Wonju 26426, Republic of Koreacs-kim@yonsei.ac.kr (C.-S.K.); 7 Central Research Laboratory, Yonsei University Wonju College of Medicine, Wonju 26426, Republic of Korea; sjh@yonsei.ac.kr; 8 Department of Surgery, Yonsei University Wonju College of Medicine, Wonju 26426, Republic of Korea

**Keywords:** acute kidney injury, multiorgan distress syndrome, biomarker, mitochondrial DNA

## Abstract

*Background and Objectives*: Critically ill surgical patients are susceptible to various postoperative complications, including acute kidney injury (AKI) and multiorgan distress syndrome (MODS). These complications intensify patient suffering and significantly increase morbidity and mortality rates. This study aimed to identify the biomarkers for predicting AKI and MODS in critically ill surgical patients. *Materials and Methods*: We prospectively enrolled critically ill surgical patients admitted to the intensive care unit via the emergency department between July 2022 and July 2023. A total of 83 patients were recruited, and their data were used to analyze MODS. Three patients who showed decreased creatinine clearance at the initial presentation were excluded from the analysis for AKI. Patient characteristics and laboratory parameters including white blood cell (WBC) count, neutrophil count, delta neutrophil index, urine and serum β2-microglobulin, and urine serum mitochondrial DNA copy number (mtDNAcn) were analyzed to determine the reliable biomarker to predict AKI and MODS. *Results*: The following parameters were independently correlated with MODS: systolic blood pressure (SBP), initial neutrophil count, and platelet count, according to a logistic regression model. The optimal cut-off values for SBP, initial neutrophil count, and platelet count were 113 mmHg (sensitivity 66.7%; specificity 73.9%), 8.65 (X^3^) (10^9^/L) (sensitivity 72.2%; specificity 64.6%), and 195.0 (X^3^) (10^9^/L) (sensitivity 66.7%; specificity 81.5%), respectively. According to the logistic regression model, diastolic blood pressure (DBP) and initial urine mtDNAcn were independently correlated with AKI. The optimal cut-off value for DBP and initial urine mtDNAcn were 68.5 mmHg (sensitivity 61.1%; specificity 79.5%) and 1225.6 copies/μL (sensitivity 55.6%; specificity 95.5%), respectively. *Conclusions*: SBP, initial neutrophil count, and platelet count were independent predictors of MODS in critically ill patients undergoing surgery. DBP and initial urine mtDNAcn levels were independent predictors of AKI in critically ill surgical patients. Large-scale multicenter prospective studies are needed to confirm our results.

## 1. Introduction

Critically ill surgical patients are susceptible to various postoperative complications, including acute kidney injury (AKI) and multiorgan distress syndrome (MODS). These complications not only intensify patient suffering, but also significantly increase morbidity and mortality rates, compelling the medical community to explore innovative approaches for their early identification and management [1,2]. Biomarkers have emerged as promising candidates for addressing this clinical need.

Early detection of AKI and MODS is pivotal for informed clinical decision making and optimized patient care. Recently, the biological relevance of mitochondrial DNA (mtDNA) in predicting adverse outcomes has been increasingly recognized, particularly in patients in the intensive care unit (ICU) [3,4,5,6]. Mitochondrial integrity plays a pivotal role in AKI pathophysiology. In various forms of AKI, early pathological alterations are evident within the renal tubular epithelium, including reduced mitochondrial abundance, organelle swelling, and fragmentation [7]. The impaired state of these mitochondria causes kidney damage by producing detrimental reactive oxygen species and releasing mtDNA. The release of mtDNA can activate innate immune responses via the Toll-like receptor-9 (TLR9) pathway [8]. Moreover, mtDNA acts as a damage-associated molecular pattern initiator [9] that can drive molecular processes leading to inflammatory responses and organ injuries [9,10,11].

β2-microglobulin (β2-MG) has also been investigated recently as a predictive marker, in particular for AKI [12,13]. β2-MG is released into circulation at a constant rate, freely filtered by the glomeruli, and completely reabsorbed and catabolized in the renal tubules. Serum β2-MG levels are independent of muscle mass and start increasing early during kidney failure. Because of these properties, serum β2-MG has been proposed as a candidate marker to assess kidney function. Tubular injury leads to decreased reabsorption of β2-MG and tubular enzymes, leading to elevated urinary concentrations. In addition, a few studies reported that β2-MG was associated with severe inflammation [14,15].

Based on these findings, we hypothesized that urine mtDNA, circulating cell-free mtDNA levels, serum β2-MG, and urinary β2-MG would be associated with AKI and MODS and would improve risk prediction in critically ill surgical patients. Although many studies have investigated the effectiveness of mtDNA as a predictive marker, studies conducted on patients admitted to the intensive care unit because of acute surgical illnesses are rare.

Therefore, this study aimed to test whether circulating cell-free mtDNA, urinary mtDNA copy number (mtDNAcn), serum β2-MG, and urinary β2-MG are useful as biomarkers in critically ill surgical patients. We also investigated other well-known biomarkers, including the delta neutrophil index (DNI), white blood cell (WBC) count, and neutrophil count because of the scarcity of studies on critically ill surgical patients.

## 2. Materials and Methods

The protocol of this study was registered in clinicaltrials.gov (NCT05458063).

### 2.1. Patient Selection

This was a prospective, observational study. This study was approved by the Ethics Committee of Wonju Severance Christian hospital (Institutional Review Board No. CR322053). We prospectively enrolled critically ill surgical patients admitted to the ICU via emergency department between July 2022 and July 2023. The exclusion criteria were as follows: (1) age < 18 years; (2) pregnancy; (3) death at initial presentation; (4) history of underlying chronic renal disease; and (5) patients with a previous history of AKI. A total of 120 patients were screened initially. Among them, 83 patients were recruited and their data were used to analyze MODS. Three patients with decreased creatinine clearance were excluded from the AKI risk factor analysis.

### 2.2. Data Collection and Definition

After obtaining written informed consent, background clinical information and the presence of other comorbid conditions were recorded. The following parameters were recorded prospectively: age; sex; vital signs at the initial presentation including systolic blood pressure (SBP), diastolic blood pressure (DBP), pulse rate (PR), and body temperature (BT); and laboratory parameters at the initial presentation including DNI, WBC, neutrophil, and platelet counts, and levels of creatinine, serum β2-MG, urine β2-MG, hemoglobin, the international normalized ratio (INR), c-reactive protein (CRP), lactate, serum mtDNAcn, and urine mtDNAcn. In addition, DNI, WBC count, neutrophil count, serum and urine β2-MG levels, and serum and urine mtDNAcn on first, second, and third days after admission were recorded to observe daily changes. The sequential organ failure assessment (SOFA) score was calculated and collected prospectively during the ICU stay. After the patients were discharged, outcomes, including AKI, MODS, and mortality, were recorded. MODS was defined as a SOFA score of 6 or higher on two or more consecutive days, at least 48 h after emergency department admission [16]. AKI was defined according to the Acute Kidney Injury Network (AKIN) criteria [17].

### 2.3. Blood and Urine Sampling, Preparation, and Storage

Blood samples were drawn and transferred into ethylenediamine tetraacetic acid (EDTA)-coated blood collection tubes at the initial presentation. Blood samples were collected at 24, 48, and 72 h after the first collection. The blood samples were sent to the central laboratory within 2 h after every venipuncture, and the EDTA tubes were centrifuged with 1300× *g* for 10 min at −4 °C. After first centrifugation, 1500 μL supernatant was collected and transferred into a 1.5 mL microtube. The microtube was centrifuged with 4000× *g* for 10 min at −4 °C, and 1000 μL supernatant was transferred into a new sterile 1.5 mL microtube, and stored at −80 °C in the freezer. Urine samples were drawn and transferred into urine tubes at the initial presentation and at 24, 48, and 72 h after the first collection. The urine samples were also sent to the central laboratory within 2 h after collection, and 1500 μL urine was collected, and transferred into a 1.5 mL microtube. The microtube was centrifuged with 4000× *g* for 10 min at −4 °C, and 1000 μL supernatant was transferred into a 1.5 mL microtube, and stored at −80 °C in the freezer until analysis. Freeze–thaw cycles were avoided to reduce the phenomenon of DNA fragmentation, and the extracts were not retained for >3 months at −80 °C.

### 2.4. DNA Isolation from Plasma and Urine

DNA extraction from plasma and urine was performed using a DNA mini kit (#51306; Qiagen, Hilden, Germany) following the manufacturer’s protocol. We incubated samples with lysis buffer (included in the kit) and proteinase K at 56 °C for 10 min. After adding ethanol (96–100%), the mixture was applied to a mini spin column (included in the kit) and centrifuged, and the filtrate was removed. The pellet in the mini-spin column was washed twice with two washing buffers (included in the kit). DNA was eluted in 50 μL of distilled water.

### 2.5. Primers and Quantitative Polymerase Chain Reaction (qPCR)

DNA was extracted from 200 µL (sample volume) plasma and urine using the QIAamp DNA mini kit (#51306; Qiagen, Germany). Purified DNA was eluted in 50 µL (elution volume) distilled water. The mtDNAcn was measured by a SYBR Green dye-based qPCR assay using Quantstudio 6 Flex real time PCR (Applied Biosystems, Waltham, MA, USA). The primer sequences were as follows: H-Mito Forward: 5′-CACTTTCCACACAGACATCA-3′, Reverse: 5′-TGGTTAGGCTGGTGTTAGGG-3′ [18]. A dilution series from 1 × 10^8^ copies/μL to 1 × 10^2^ copies/μL consisting of the purified PCR product from a healthy control participant, not participating in the present study, was constructed and used to create a standard curve. Concentrations were converted to copy number using the formula: mol/g 6 molecules/mol = molecules/g via a DNA copy number calculator (http://cels.uri.edu/gsc/cndna.html (accessed on 16 May 2023); University of Rhode Island Genomics and Sequencing Center).

**Figure 1:**
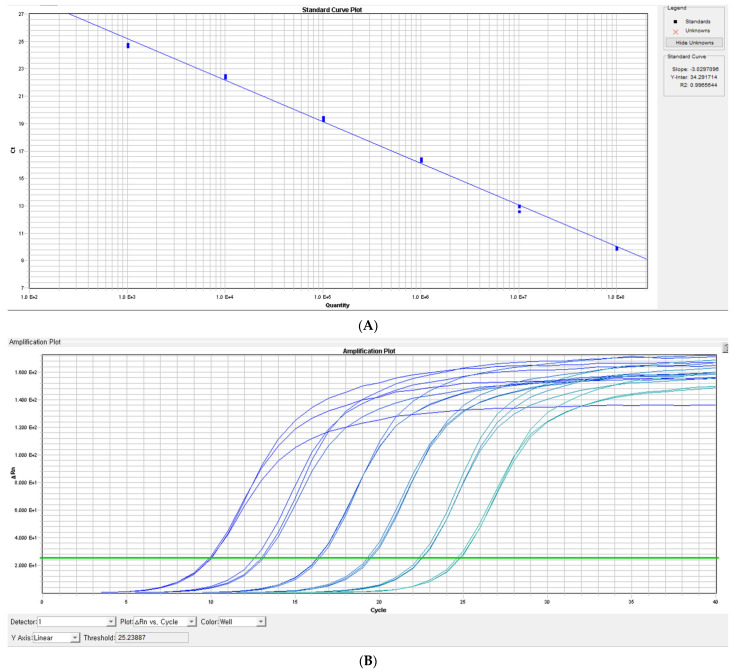

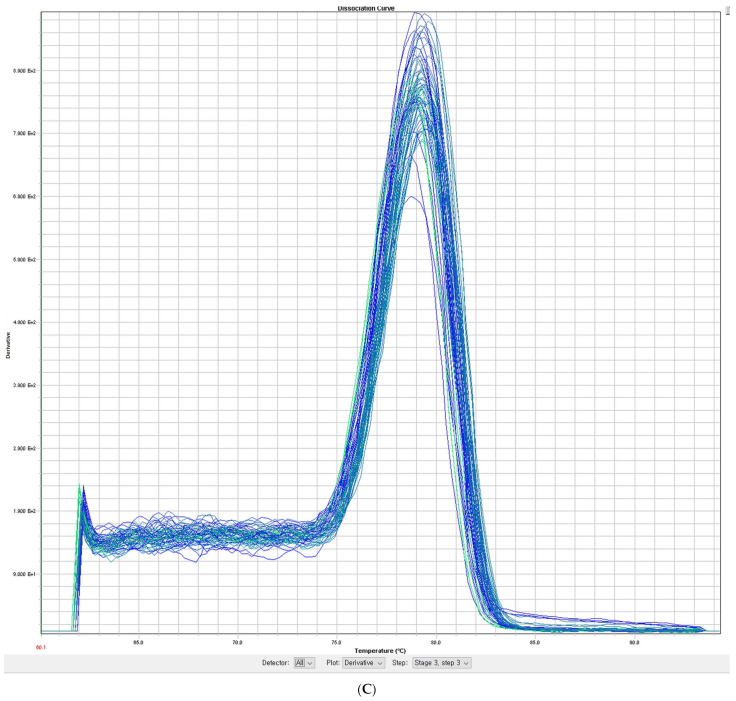
(**A**) representative standard curve. The linear blue line represents the standard curve used for measuring standard DNAs. (**B**) amplification plot. The bright blue line on the far left indicates the amplification curve of standard DNA at a concentration of 1 × 10^8^ copies/ µL, and the green line at the far right indicates the amplification curve of standard DNA at a concentration of 1 × 10^2^ copies/µL. (**C**) dissociation curve for standard DNA.

The thermal profile for detecting mtDNA using Quantinova SYBR Green PCR kit (Qiagen, Germany) was analyzed as follows: an initiation step for 2 min at 60 °C was followed by an initial denaturation step for 10 min at 95 °C and a further step consisting of 40 cycles for 20 s at 95 °C and for 10 s at 60 °C. The representative standard curves, dissociation curves, and amplification plots are shown in Figure 1.

All samples were analyzed in triplicate, and a no-template control was included in each analysis. The mtDNA levels in all plasma analyses were expressed in copies per microliter of plasma based on the following calculations [19]:

$$Copy\;Number\;per\;\mu L=\frac{Copy\;number\;per\;2\;\mu L\;reaction\times Elution\;volume\;\left(\mu L\right)\;}{qPCR\;reaction\;volume\;\left(\mu L\right)}\times \frac{1}{Sample\;volume\;\left(\mu L\right)}$$

### 2.6. Statistical Analysis

Statistical analyses were performed using R statistical software (version 4.1.0; R Foundation for Statistical Computing, Vienna, Austria). Continuous variables are presented as the mean and standard deviation or median and range, and a comparative analysis was conducted using the independent-samples *t*-test. The Mann–Whitney U-test was performed for continuous variables that were non-normally distributed. Chi-squared and Fisher’s exact tests were used for comparative analysis of categorical variables. Multivariate analysis was performed using logistic regression to identify independent risk factors. A receiver-operating characteristic (ROC) curve was constructed and the Youden index method was used to determine the optimal cut-off values for predicting mortality. Statistical significance was set at *p*-value < 0.05.

## 3. Results

### 3.1. Baseline Characteristics in the Enrolled Patients

Mean age in all patients was 63.3 ± 17.3 years, and the male:female ratio was 56:27. A total of 30 patients (36.1%) had hypertension and 18 patients (21.7%) had diabetes mellitus. The most common diagnosis was pan-peritonitis (41 patients [49.4%]), followed by hemoperitoneum (16 patients [19.2%]). Surgical treatment was performed in 76 patients (91.6%) (Table 1).

### 3.2. Patient Characteristics between Patients without and with MODS

Mean age (62.0 ± 18.4 vs. 68.0 ± 11.7 years, *p* = 0.317) and the ratio of male sex (42 [64.6%] vs. 14 [77.8%], *p* = 0.441) were not significantly different between both groups. SBP (126.1 ± 26.5 vs. 98.2 ± 31.5 mmHg, *p* < 0.001) and DBP (70.6 ± 16.4 vs. 55.2 ± 15.4 mmHg, *p* = 0.001) were significantly lower in patients with MODS. Initial DNI (4.1 ± 8.4% vs. 9.8 ± 12.2%, *p* = 0.016), WBC (12.8 ± 5.6 vs. 10.0 ± 4.9 (X^3^) (10^9^/L), 0.046), neutrophil (10.8 ± 5.2 vs. 7.8 ± 3.8 (X^3^) (10^9^/L), *p* = 0.028), creatinine (0.8 ± 0.3 vs. 1.2 ± 0.3 mg/dL, *p* < 0.001), serum β2-MG (2.5 ± 2.0 vs. 4.2 ± 4.1 mg/L, *p* = 0.008), hemoglobin (13.1 ± 2.3 vs. 11.8 ± 1.9 g/dL, *p* = 0.026), platelet (261.0 ± 107.3 vs. 188.9 ± 81.6 (X^3^) (10^9^/L), *p* = 0.004), INR (1.0 ± 0.1 vs. 1.2 ± 0.2, *p* < 0.001), and lactate (2.5 ± 1.9 vs. 4.7 ± 3.9 mmol/L, *p* = 0.005) were significantly different between both groups. Initial urine mtDNAcn was significantly higher in patients with MODS (1067.5 ± 1575.8 vs. 3800.4 ± 7298.9 copies/μL, *p* = 0.032). The highest SOFA score was significantly higher in patients with MODS (2.0 ± 1.6 vs. 8.1 ± 2.0, *p* < 0.001). Hospital duration of stay (DOS) (15.8 ± 10.1 vs. 28.7 ± 22.0 days, *p* = 0.008) and ICU DOS (3.5 ± 3.7 vs. 9.1 ± 7.7 days, *p* = 0.001) were significantly higher in patients with MODS. Mortality was more frequent in patients with MODS (0 [0%] vs. 5 [27.8%], *p* < 0.001) (Table 2).

### 3.3. Independent Risk Factors to Predict MODS in Critically Ill Surgical Patients

A logistic regression model was performed to determine independent risk factors, including age, sex, SBP, DBP, initial DNI, WBC, neutrophil, creatinine, serum β2-MG, hemoglobin, platelet, INR, lactate, and initial urine mtDNAcn. The following were independently correlated with MODS: SBP (odds ratio [OR] 0.97, 95% confidence interval [CI] 0.94–1.00, *p* = 0.025], initial neutrophil count [OR 0.84, 95% CI 0.72–0.98, *p* = 0.024], and initial platelet count [OR 0.99, 0.98–1.00, *p* = 0.036] (Table 3).

### 3.4. Optimal Cut-Off Value of SBP and Initial Neutrophil and Platelet Counts to Predict MODS in Critically Ill Surgical Patients

**Figure 2:**
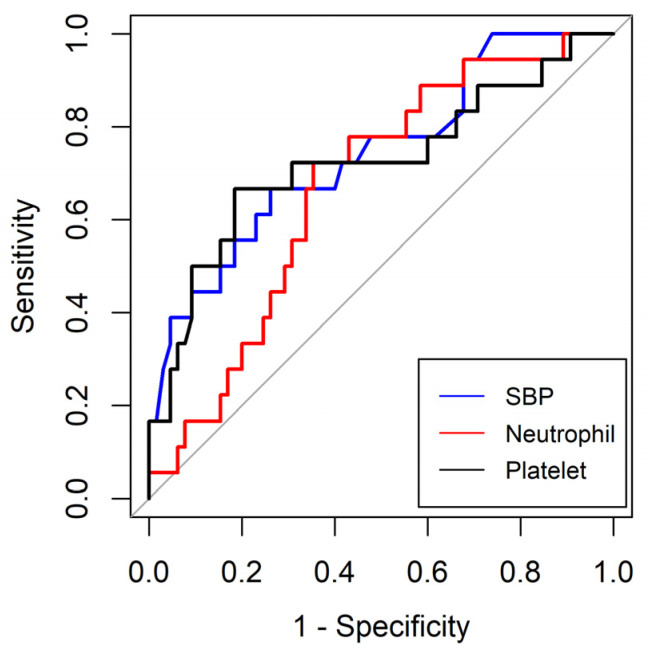
Receiver-operating characteristics curves for the systolic blood pressure and initial neutrophil and platelet counts between the patients with and without multiorgan distress syndrome. SBP, systolic blood pressure.

The ROC curves of SBP and initial neutrophil and platelet counts were calculated to predict MODS in critically ill patients undergoing surgery. The areas under the curve (AUC) of SBP and initial neutrophil and platelet counts were 0.750 (95% CI 0.606–0.877), 0.670 (95% CI 0.539–0.801), and 0.724 (0.570–0.877), respectively. The optimal cut-off values for SBP and initial neutrophil and platelet counts were 113 mmHg (sensitivity 66.7%; specificity 73.9%), 8.65 (X^3^) (10^9^/L) (sensitivity 72.2%; specificity 64.6%), and 195.0 (X^3^) (10^9^/L) (sensitivity 66.7%; specificity 81.5%), respectively (Table 4 and Figure 2).

### 3.5. Patient Characteristics between Patients without and with AKI

Mean age (59.4 ± 17.9 vs. 66.2 ± 15.5 years, *p* = 0.072) and the ratio of male sex (30 [68.2%] vs. 24 [66.7%], *p* = 1.000) were not significantly different between both groups. SBP (127.2 ± 19.3 vs. 112.8 ± 37.9 mmHg, *p* = 0.044) and DBP (74.4 ± 13.8 vs. 58.6 ± 17.8 mmHg, *p* < 0.001) were significantly lower in patients with AKI. Initial DNI (2.7 ± 4.8% vs. 7.3 ± 11.0%, *p* = 0.035), WBC (15.1 ± 13.4 vs. 11.6 ± 5.9 (X^3^) (10^9^/L), *p* = 0.085), neutrophil (11.2 ± 4.7 vs. 9.3 ± 5.2 (X^3^) (10^9^/L), *p* = 0.045), creatinine (0.8 ± 0.2 vs. 1.0 ± 0.4 mg/dL, *p* = 0.006), serum β2-MG (2.1 ± 1.4 vs. 3.4 ± 3.4 mg/L, *p* = 0.020), hemoglobin (13.4 ± 2.1 vs. 12.2 ± 2.3 g/dL, *p* = 0.011), platelet (263.6 ± 93.6 vs. 222.6 ± 120.6 (X^3^) (10^9^/L), *p* = 0.016), INR (1.0 ± 0.1 vs. 1.1 ± 0.2, *p* = 0.007), and lactate (2.2 ± 1.5 vs. 3.9 ± 3.3 mmol/L, *p* = 0.004) were significantly different between both groups. Initial urine mtDNAcn was significantly higher in patients with AKI (561.4 ± 349.9 vs. 3001.8 ± 5424.5 copies/μL, *p* = 0.001). The highest SOFA score was significantly higher in patients with AKI (2.2 ± 2.0 vs. 4.5 ± 3.7, *p* = 0.004). Hospital DOS (15.9 ± 14.7 vs. 21.6 ± 14.3 days, *p* = 0.007) and ICU DOS (3.1 ± 3.7 vs. 6.5 ± 6.4 days, *p* = 0.010) were significantly higher in patients with AKI. MODS (4 [9.1%] vs. 14 [38.9%], *p* = 0.004) and mortality were more common in patients with AKI (0 [0%] vs. 5 [13.9%], *p* = 0.037) (Table 5).

### 3.6. Independent Risk Factors to Predict AKI in Critically Ill Surgical Patients

A logistic regression model was performed to determine independent risk factors, including age, sex, SBP, DBP, initial DNI, neutrophil, creatinine, serum β2-MG, hemoglobin, platelet, INR, lactate, and initial urine mtDNAcn. The following were independently correlated with AKI: DBP (OR 0.90, 95% CI 0.84–0.96, *p* = 0.001) and initial urine mtDNAcn (OR 1.00, 95% CI 1.00–1.00, *p* = 0.010) (Table 6).

### 3.7. Optimal Cut-Off Value of DBP and Initial Urine mtDNAcn to Predict AKI in Critically Ill Surgical Patients

**Figure 3:**
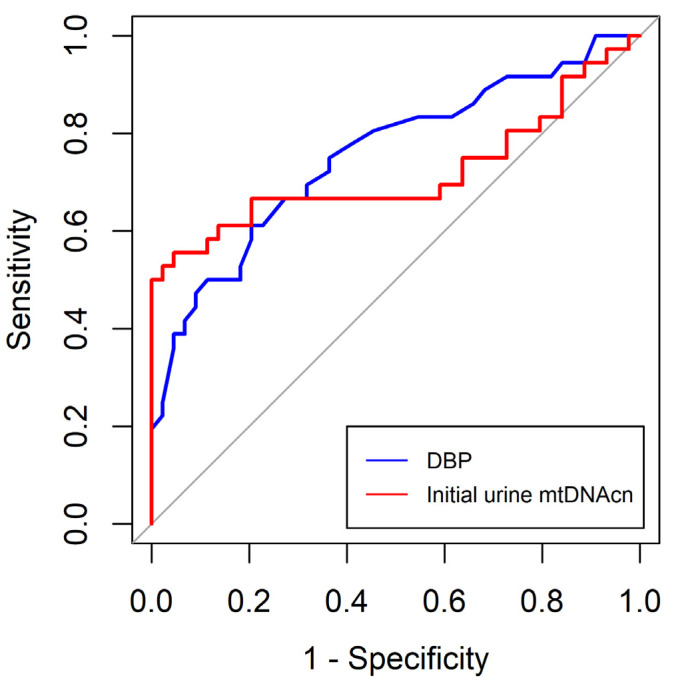
Receiver-operating characteristics curves for the diastolic blood pressure and initial urine mitochondrial DNA copy number between the patients with and without acute kidney injury. DBP, diastolic blood pressure; mtDNAcn, mitochondrial DNA copy number.

The ROC curves of DBP and initial urine mtDNAcn were calculated to predict AKI in critically ill surgical patients. The AUC of DBP and initial urine mtDNAcn were 0.755 (95% CI 0.647–0.864) and 0.718 (95% CI 0.590–0.845), respectively. The optimal cut-off values for DBP and initial urine mtDNAcn were 68.5 mmHg (sensitivity 61.1%; specificity 79.5%) and 1225.6 (sensitivity, 55.6%; specificity, 95.5%), respectively (Table 7 and Figure 3).

## 4. Discussion

In this study, we found that SBP and initial neutrophil and platelet counts were independently associated with MODS in critically ill patients undergoing surgery. The optimal cut-off values for SBP and initial neutrophil and platelet counts were 113 mmHg, 8.65 (X^3^)(10^9^/L), and 195.0 (X^3^)(10^9^/L), respectively. In addition, DBP and initial urine mtDNAcn levels were independently associated with AKI in critically ill surgical patients. The optimal cut-off values for DBP and initial urine mtDNAcn were 68.5 mmHg and 1225.6 copies/μL, respectively.

Consistent with our findings, several studies have reported an established relationship between SBP and MODS occurrence across various clinical contexts [20,21]. This underscores the importance of maintaining optimal perfusion and effectively managing blood pressure in critically ill patients. Transient episodes of hypotension can precipitate inadequate oxygen delivery to vital organs, initiating a cascade of events that may ultimately culminate in MODS. Early recognition and prompt management of hypotension are imperative for preventing severe medical complications.

Polymorphonuclear leukocytes, monocytes, macrophages, dendritic cells, and natural killer cells are pivotal contributors to the cellular immune response following infection or trauma [22,23]. Neutrophils are attracted to the site of injury under the influence of cytokines, such as interleukin (IL)-8, where they actively participate in the immune response by combatting pathogens and assisting in the removal of damaged tissue. Additionally, these cells play a role in the initiation of molecules such as tumor necrosis factor (TNF)-α, IL-8, platelet-activating factor, and anaphylatoxin (C5a). This sequence of events leads to an increased inflammatory response, which triggers the activation and recruitment of polymorphonuclear leukocytes. This, in turn, contributes to the onset of the systemic inflammatory response syndrome and MODS [22,23,24]. Recruitment of polymorphonuclear cells leads to neutrophilia. This period can be considered a “vulnerability window” during which a subsequent event, often referred to as a “second hit”, can trigger the onset of MODS [24,25]. In patients who develop MODS, initial neutrophilia is followed by neutropenia following trauma or infection [26]. Therefore, patients with neutropenia at initial presentation may progress to MODS, as shown in our study.

Thrombocytopenia at initial presentation is most likely caused by the loss or consumption of platelets or sepsis in critically ill surgical patients. Thrombocytopenia in patients with sepsis occurs through several mechanisms. Platelets are activated during sepsis and adhere to the endothelial lining, leading to their sequestration and subsequent breakdown. Additionally, immune-related factors such as non-specific antibodies associated with platelets and cytokine-driven phagocytosis of platelets can also play a role in the development of thrombocytopenia in sepsis [27]. Therefore, the development of thrombocytopenia indicates progression to more serious adverse events. Similar to our results, previous studies have reported that thrombocytopenia poses an independent risk for adverse events in ICU patients [28,29].

DBP serves as an indicator of the vascular tone. A study has illustrated that elevated levels of naturally occurring inflammatory mediators, such as nitrous oxide and TNF-α, are linked to the progression of severe sepsis, shock, or even mortality [30]. These cytokines and autocrine hormones have vasodilatory properties. Patients with sepsis who exhibit a normal mean SBP and low DBP are in a state of compensated vasodilation, which precedes a more evident cardiovascular collapse. In patients with trauma, there can be an initial mild shock leading to an increase in systemic venous resistance and subsequently higher DBP. However, when severe and ongoing bleeding occurs, resulting in a loss of over 20–30% of the total blood volume, systemic venous resistance may decrease, leading to failure of vascular compensation [31]. This, in turn, causes a decline in DBP. Therefore, a reduction in DBP in patients with trauma results in a substantial loss of blood volume. In other words, a lower DBP reflects a lower effective circulating volume at initial presentation and a high prevalence of AKI.

The mtDNA has a damage-associated molecular pattern (DAMP). In a landmark study in 2004, Collins et al. [32]. found that injecting mtDNA into the joints of mice resulted in localized inflammation. Many studies have investigated the mechanism by which mtDNA affects the inflammatory cascade by acting as a DAMP. First, mtDNA is released into the cytoplasm or extracellular space via various mechanisms in various clinical settings. Various mitochondrial stresses, including bacterial and viral infections or trauma-induced cellular damage, can lead to the release of mtDNA associated with reactive oxygen species. Alternatively, activation of Bcl-2-associated X protein (BAX) and Bcl-2 homologous antagonist/killer (BAK) leads to outer mitochondrial membrane permeabilization (MOMP) and mtDNA release [33]. Pathogen-infected cells often secrete IL-1β due to inflammasome activation. A recent report by Aarreberg et al. [34] discovers a link between IL-1β secretion in infected cells, which can then activate a cyclic GMP-AMP synthase (cGAS) stimulator of interferon genes (STING)-dependent type-I interferon response in surrounding bystander cells. Interestingly, IL-1β stimulation of bystander cells increases mitochondrial mass, decreases mitochondrial membrane potential, and induces mtDNA release.

Once cytoplasmic, mtDNA released from mitochondria can also bind the DNA sensing protein cGAS that catalyzes the production of the secondary messenger 2030 cyclic GMP–AMP (2030cGAMP) from adenosine triphosphate (ATP) and guanosine-5′-triphosphate (GTP). Cyclic guanosine monophosphate-adenosine monophosphate (cGAMP) binds to the adaptor molecule STING on the endoplasmic reticulum, leading to the activation of TANK-binding kinase 1 (TBK1). Active TBK1 phosphorylates the transcription factor interferon regulatory factor 3 (IRF3) initiating a type-I interferon response [33]. Elevated levels of type-I interferons can hinder B-cell reactions, possibly inducing the generation of immunosuppressive substances. Additionally, these elevated concentrations diminish the ability of macrophages to respond effectively to interferon-γ activation [35].

Cytoplasmic mtDNA can enter the extracellular environment through necroptosis or platelet aggregation [36]. The released circulating mtDNA is detected by the TLR9 located on the neutrophil surface, which activates p38 mitogen-activated protein kinase (MAPK). Subsequently, diverse transcriptional factors including nuclear factor kappa-light-chain-enhancer of activated B cells is activated via MAPK, and thereby a cellular response is induced. Cellular responses include the expression of cytokines or adhesion molecules that accelerate inflammation and diapedesis of immune effector cells. In addition, released IL-1β also contributes to systemic inflammatory responses.

Therefore, we hypothesized that serum mtDNAcn level may be a predictor of MODS. However, we did not observe any difference in serum mtDNAcn levels between patients with and without MODS. Nakashira et al. [5] reported that circulating mtDNA levels were associated with mortality in medical ICU patients. Harrington et al. [36] reported that 11 out of 16 studies reported a significant association between circulating mtDNA levels and mortality among critically ill patients in their systematic review. These discrepancies between studies on critically ill patients, including that of our study, although our outcome was not mortality, may be due to the indirectness of the participants and small number of participants.

Mitochondria are pivotal contributors to AKI, serving a dual function as the primary energy source for cells and a crucial controller of cell death processes. Initially, alterations in the mitochondrial structure were detected in AKI cases. A primary cause of AKI is ischemia, which leads to a reduction in the number of mitochondria and causes structural changes in these organelles, characterized by swelling and loss of the inner mitochondrial membrane cristae. These changes occur because of diminished ATP levels and a decline in membrane potential [37]. Furthermore, the opening of mitochondrial permeability transition pores (mPTP) is a pivotal event resulting from mitochondrial swelling and dysfunction. This event significantly contributes to AKI progression by releasing proapoptotic substances such as cytochrome c, which can trigger apoptosis in renal cells [38]. Second, an imbalance in the regulation of mitochondrial fission and fusion has been linked to AKI progression. In this context, dynamin-related protein 1, which is responsible for controlling mitochondrial fission, exhibits swift activation, whereas mitofusin and optic atrophy 1, which oversee mitochondrial fusion, experience a reduction in their levels during AKI. This process leads to fragmentation of mitochondria [39]. Third, the released mtDNA has the ability to bind to the DNA-sensing protein cGAS, triggering a sequence of events, as described earlier. Fourth, oxidative stress produces mitochondrial reactive oxygen species, which can destroy renal cells [40]. Finally, the release of mtDNA as one of DAMPs from mitochondria into the extracellular space accelerates a vicious spiral of renal cellular damage [41]. Urinary mtDNA levels can be elevated because of AKI-related mitochondrial dysfunction. Hu et al. [41] reported that urinary mtDNA levels were associated with new-onset AKI in surgical ICU patients, which is consistent with our results. However, Whitaker et al. [42] showed that urinary mtDNA levels did not differ between AKI and non-AKI groups after cardiac surgery. Additional research is needed to gain a more comprehensive understanding of the predictive potential of urinary mtDNA for AKI as variations and inconsistencies persist among studies.

Although serum β2-MG was not an independent predictor of AKI and MODS (*p* = 0.053) in the multivariate analysis in this study, it could be a candidate of predictors for AKI and MODS. β2-MG is filtered by glomeruli and subsequently reabsorbed in the proximal tubule. Serum β2-MG is independent of muscle mass and starts increasing early in kidney failure. β2-MG is present in all nucleated cells [43] and is not affected by sex or age [44]. These properties may make β2-MG an ideal endogenous biomarker for estimating the glomerular filtration rate. Tubular injury leads to decreased reabsorption of β2-MG and tubular enzymes, leading to elevated urinary concentrations. Thus, these markers may act as functional markers of tubular damage and fibrosis. In addition, β2-MG may be related to inflammation. Although not fully understood, β2-MG may induce IL-1β and IL-18 release by macrophages in a caspase-1- and nod-like receptor family pyrin-domain-containing 3-dependent manner [45]. Further investigation is necessary to determine the association between β2-MG and adverse outcomes.

Our study has several limitations. First, our sample size and the number of patients with MODS were small (*n* = 18). Second, several methodological factors may have influenced our results. For example, although we prepared samples within 2 h of collection, we cannot rule out the possibility that slight differences in the timing of sample collection could influence the associations within the groups (AKI vs. no AKI, MODS vs. no MODS). Moreover, slight differences in mtDNA measurements can occur throughout the process, from sample collection to qPCR. Third, although we considered many variables for the multivariate analysis, some possible factors could have been missed. Finally, our study included surgical patients admitted to the ICU, both trauma and non-trauma patients. The mechanism of MODS development may differ between patients, depending on whether it is related to infection or sterile inflammation. This may have influenced the results of this study.

## 5. Conclusions

Despite these limitations, our study is meaningful because it is a rare prospective observational study that comprehensively analyzed biomarkers to predict MODS and AKI in critically ill surgical patients. SBP, initial neutrophil count, and platelet count were independent predictors of MODS in critically ill patients. In addition, we found that DBP and initial urine mtDNAcn levels were independent predictors of AKI in critically ill surgical patients. Large-scale multicenter prospective studies are needed to confirm our results.

## Figures and Tables

**Table 1:** Baseline characteristics in the enrolled patients.

Variables	*n* = 83 (%)
Age	63.3 ± 17.3
Male:Female	56:27
Known past history	
Hypertension	30 (36.1)
Diabetes mellitus	18 (21.7)
Cerebrovascular disorder	7 (8.4)
Liver disease	3 (3.6)
Respiratory disease	7 (8.4)
Diagnosis	
Cervical spine fracture	1 (1.2)
Diaphragmatic rupture	1 (1.2)
Femur fracture	3 (3.6)
Fournier’s gangrene	1 (1.2)
Hemoperitoneum	16 (19.2)
Hemopneumothorax	1 (1.2)
Ischemic enteritis	3 (3.6)
Intestinal obstruction	3 (3.6)
Panperitonitis	41 (49.4)
Popliteal artery rupture	1 (1.2)
Retroperitoneal hemorrhage	3 (3.6)
Small bowel strangulation	8 (9.6)
Thoracic spine fracture	1 (1.2)
Procedure	
Conservative management with observation only	4 (4.8)
Angioembolization	3 (3.6)
Surgery	76 (91.6)

**Table 2:** Patient characteristics between patients without and with multiorgan distress syndrome.

	No MODS (*n* = 65) (%)	MODS (*n* = 18) (%)	*p*-Value
Age (years)	62.0 ± 18.4	68.0 ± 11.7	0.317
Male sex	42 (64.6)	14 (77.8)	0.441
Known past history			
Hypertension	23 (35.4)	7 (38.9)	0.841
Diabetes mellitus	14 (21.5)	4 (22.2)	1.000
Cerebrovascular disorder	4 (6.2)	3 (16.7)	0.148
Liver disease	1 (1.5)	2 (11.1)	0.105
Respiratory disease	6 (9.2)	1 (5.6)	1.000
SBP (mmHg)	126.1 ± 26.5	98.2 ± 31.5	<0.001
DBP (mmHg)	70.6 ± 16.4	55.2 ± 15.4	0.001
PR (/min)	93.9 ± 45.0	96.7 ± 28.5	0.133
BT (°C)	36.8 ± 0.8	36.5 ± 1.4	0.205
Initial laboratory findings			
DNI (%)	4.1 ± 8.4	9.8 ± 12.2	0.016
WBC (X^3^) (10^9^/L)	12.8 ± 5.6	10.0 ± 4.9	0.046
Neutrophil (X^3^) (10^9^/L)	10.8 ± 5.2	7.8 ± 3.8	0.028
Creatinine (mg/dL)	0.8 ± 0.3	1.2 ± 0.3	<0.001
Serum β2 microglobulin (mg/L)	2.5 ± 2.0	4.2 ± 4.1	0.008
Urine β2 microglobulin (mg/L)	5.9 ± 11.0	4.6 ± 5.0	0.548
Hemoglobin (g/dL)	13.1 ± 2.3	11.8 ± 1.9	0.026
Platelet (X^3^) (10^9^/L)	261.0 ± 107.3	188.9 ± 81.6	0.004
INR	1.0 ± 0.1	1.2 ± 0.2	<0.001
CRP (mg/dL)	5.7 ± 9.1	6.0 ± 10.1	0.958
Lactate (mmol/L)	2.5 ± 1.9	4.7 ± 3.9	0.005
Serum mtDNAcn (copies/μL)	716.0 ± 1357.6	1311.8 ± 1872.6	0.260
Urine mtDNAcn (copies/μL)	1067.5 ± 1575.8	3800.4 ± 7298.9	0.032
SOFA score	2.0 ± 1.6	8.1 ± 2.0	<0.001
Hospital DOS	15.8 ± 10.1	28.7 ± 22.0	0.008
ICU DOS	3.5 ± 3.7	9.1 ± 7.7	0.001
Mortality	0 (0.0%)	5 (27.8%)	<0.001

MODS, multiorgan distress syndrome; SBP, systolic blood pressure; DBP, diastolic blood pressure; PR, pulse rate; BT, body temperature; DNI, delta neutrophil index; WBC, white blood cells; INR, international normalized ratio; CRP, C-reactive protein; mtDNAcn, mitochondrial DNA copy number; SOFA, sequential organ failure assessment; ICU, intensive care unit; DOS, duration of stay.

**Table 3:** Multivariate analysis using a logistic regression model to predict multiorgan distress syndrome.

	Odds Ratio (95% CI)	*p*-Value
SBP (mmHg)	0.97 (0.94–1.00)	0.025
Initial neutrophil (X^3^) (10^9^/L)	0.84 (0.72–0.98)	0.024
Initial creatinine (mg/dL)	8.27 (0.70–97.12)	0.093
Initial serum β2 microglobulin (mg/L)	1.30 (1.00–1.69)	0.053
Initial platelet (X^3^) (10^9^/L)	0.99 (0.98–1.00)	0.036

CI, confidence interval; SBP, systolic blood pressure.

**Table 4:** Systolic blood pressure and initial neutrophil and platelet count characteristics as independent predictors of multiorgan distress syndrome.

	Optimal Cut-Off Value	Sensitivity %	Specificity %	AUC (95% CI)
SBP (mmHg)	113	66.7	73.9	0.750 (0.606–0.877)
Initial neutrophil count (X^3^) (10^9^/L)	8.65	72.2	64.6	0.670 (0.539–0.801)
Initial platelet count (X^3^) (10^9^/L)	195	66.7	81.5	0.724 (0.570–0.877)

AUC, area under the curve; CI, confidence interval; SBP, systolic blood pressure.

**Table 5:** Patient characteristics between patients without and with acute kidney injury.

	No AKI (*n* = 44) (%)	AKI (*n* = 36) (%)	*p*-Value
Age (years)	59.4 ± 17.9	66.2 ± 15.5	0.072
Male sex	30 (68.2)	24 (66.7)	1.000
Known past history			
Hypertension	13 (29.5)	15 (41.7)	0.371
Diabetes mellitus	8 (18.2)	9 (25.0)	0.641
Cerebrovascular disorder	3 (6.8)	3 (8.3)	1.000
Liver disease	2 (4.5)	1 (2.8)	1.000
Respiratory disease	0 (0.0)	1 (2.8)	1.000
SBP (mmHg)	127.2 ± 19.3	112.8 ± 37.9	0.044
DBP (mmHg)	74.4 ± 13.8	58.6 ± 17.8	<0.001
PR (/min)	88.0 ± 26.2	100.9 ± 56.0	0.183
BT (°C)	36.8 ± 1.0	36.6 ± 0.9	0.276
Initial laboratory findings			
DNI (%)	2.7 ± 4.8	7.3 ± 11.0	0.035
WBC (X^3^) (10^9^/L)	15.1 ± 13.4	11.6 ± 5.9	0.085
Neutrophil (X^3^) (10^9^/L)	11.2 ± 4.7	9.3 ± 5.2	0.045
Creatinine (mg/dL)	0.8 ± 0.2	1.0 ± 0.4	0.006
Serum β2 microglobulin (mg/L)	2.1 ± 1.4	3.4 ± 3.4	0.020
Urine β2 microglobulin (mg/L)	6.2 ± 10.6	5.3 ± 9.9	0.306
Hemoglobin	13.4 ± 2.1	12.2 ± 2.3	0.011
Platelet (X^3^) (10^9^/L)	263.6 ± 93.6	222.6 ± 120.6	0.016
INR	1.0 ± 0.1	1.1 ± 0.2	0.007
CRP (mg/dL)	5.0 ± 8.8	5.4 ± 9.0	0.815
Lactate (mmol/L)	2.2 ± 1.5	3.9 ± 3.3	0.004
Serum mtDNAcn (copies/μL)	532.6 ± 812.3	1251.4 ± 2027.6	0.158
Urine mtDNAcn (copies/μL)	561.4 ± 349.9	3001.8 ± 5424.5	0.001
SOFA score	2.2 ± 2.0	4.5 ± 3.7	0.004
Hospital DOS	15.9 ± 14.7	21.6 ± 14.3	0.007
ICU DOS	3.1 ± 3.7	6.5 ± 6.4	0.010
MODS	4 (9.1)	14 (38.9)	0.004
Mortality	0 (0.0)	5 (13.9)	0.037

AKI, acute kidney injury; SBP, systolic blood pressure; DBP, diastolic blood pressure; PR, pulse rate; BT, body temperature; DNI, delta neutrophil index; WBC, white blood cells; INR, international normalized ratio; CRP, C-reactive protein; mtDNAcn, mitochondrial DNA copy number; SOFA, sequential organ failure assessment; ICU, intensive care unit; DOS, duration of stay; MODS, multiorgan distress syndrome.

**Table 6:** Multivariate analysis using a logistic regression model to predict acute kidney injury.

	Odds Ratio (95% CI)	*p*-Value
SBP (mmHg)	1.04 (1.00–1.07)	0.059
DBP (mmHg)	0.90 (0.84–0.96)	0.001
Initial neutrophil (X^3^) (10^9^/L)	0.90 (0.79–1.03)	0.127
Initial urine mtDNAcn (copies/μL)	1.00 (1.00–1.00)	0.010

CI, confidence interval; SBP, systolic blood pressure; DBP, diastolic blood pressure; mtDNAcn, mitochondrial DNA copy number.

**Table 7:** Diastolic blood pressure and initial urine mitochondrial DNA copy number as independent predictors of acute kidney injury.

	Optimal Cut-Off Value	Sensitivity %	Specificity %	AUC (95% CI)
DBP (mmHg)	68.5	61.1	79.5	0.755 (0.647–0.864)
Initial urine mtDNAcn (copies/μL)	1225.6	55.6	95.5	0.718 (0.590–0.845)

AUC, area under the curve; DBP, diastolic blood pressure; mtDNAcn, mitochondrial DNA copy number.

## Data Availability

Data can be made available upon request.
